# Interleukin-17 promotes the production of underglycosylated IgA1 in DAKIKI cells

**DOI:** 10.1080/0886022X.2017.1419972

**Published:** 2018-01-04

**Authors:** Jia-Ru Lin, Ji Wen, Hui Zhang, Li Wang, Fang-Fang Gou, Man Yang, Jun-Ming Fan

**Affiliations:** a Department of Nephrology, The Affiliated Hospital of Southwest Medical University, Luzhou City, Sichuan Province, China;; b Department of Nephrology, The Affiliated Chinese Medicine Hospital of Southwest Medical University, Luzhou City, Sichuan Province, China;; c Department of Nephrology, West China Hospital of Sichuan University, Chengdu City, Sichuan Province, China;; d State Key Laboratory of Biotherapy of Human Disease, West China Hospital, Sichuan University, Chengdu City, Sichuan Province, China;; e Department of Central Service, West China Hospital of Sichuan University, Chengdu City, Sichuan Province, China

**Keywords:** Interleukin 17, IgA nephropathy, C1GALT1, Cosmc, underglycosylation, IgA1

## Abstract

**Background:** Interleukin 17 (IL-17) plays an important role in the pathogenesis of autoimmune diseases and might be associated with IgA nephropathy (IgAN). This study aimed to investigate the effect of IL-17 on autoimmune pathogenesis in IgA nephropathy.

**Methods:** DAKIKI cells were cultured and stimulated with IL-17 to perform dose-dependent and time-dependent experiments. Cell proliferation was examined by cell counting and the Cell Counting Kit-8 (CCK-8) assay. The IgA concentration and the degree of galactosylation in the supernatant were tested using ELISA and a helix aspersa (HAA) lectin binding assay, respectively. To study the mechanism of O-glycosylation, cells were stimulated with IL-17, lipopolysaccharide (LPS) or 5-azacytidine (5-AZA) + IL-17 for 48 h, and the levels of C1GALT1 and its molecular chaperone Cosmc were measured by western blot and real-time PCR.

**Results:** The cell counting and CCK-8 results suggested that B lymphocyte proliferation increased significantly with increased IL-17 concentration. IL-17 affected the quantity of IgA1 and its glycosylation status. HAA revealed that IL-17 promoted IgA1 underglycosylation. Mechanistically, the expression of C1GALT1 and Cosmc was significantly lower in cells stimulated by IL-17 or LPS than in the 5-AZA + IL-17 or the control group.

**Conclusions:** Our results suggested that IL-17 stimulates B lymphocyte to promote B-cell proliferation, which leads to increased IgA1 production *in vitro* accompanied by underglycosylation of IgA1. The molecular mechanism for the IgA1 underglycosylation induced by IL-17 was similar to that of LPS; however, 5-AZA inhibited IgA1 underglycosylation. IL-17 might participate in IgAN pathogenesis by influencing the production and glycosylation of IgA1 in B-cells.

## 1. Introduction

Immunoglobulin A nephropathy (IgAN) is the most common primary glomerulonephritis worldwide [1], especially in Southeast Asia. Approximately 30–40% of IgAN patients progress to end-stage renal disease (ESRD) within 20 years [2]. The pathophysiology of IgAN is not completely understood. IgAN is thought to be associated with immune abnormalities in T cells, which leads to abnormal signal transduction and results in an altered B-cell response [3,4].

Th17 cells are a type of T helper cell with the main function of secreting interleukin 17 (IL-17), which is a newly discovered cytokine implicated in inflammation regulation. At the same time, Th17 cells play a significant role in the development of autoimmune diseases including rheumatoid arthritis and multiple sclerosis. Matsumoto et al. observed increased levels of IL-17 excretion in the urine of patients with minimal-change nephrotic syndrome (MCNS) and IgAN when compared to non-nephrotic patients and healthy controls. In MCNS, the daily urinary IL-17 (uIL-17) excretion was increased, and there was a positive correlation between urinary protein excretion and daily uIL-17 excretion. IL-17 was also shown to stimulate the release of a number of pro-inflammatory cytokines from peripheral blood monocytes (PBM) in IgAN patients [5,6]. However, the molecular mechanisms mediating IL-17 involvement in the immune-related pathogenesis of IgAN have not been fully elucidated.

This study aimed to investigate the effect of IL-17 on the production and glycosylation of IgA1 and to explore the molecular mechanisms of aberrant IgA1 glycosylation in DAKIKI cells.

## 2. Materials and methods

### 2.1. Cell culture and experimental protocols

The surface IgA1-positive human B lymphoma cell line, DAKIKI, was obtained from ATCC (Manassas, VA) [7–9]. Cells were cultured in 75 cm^2^ flasks in RPMI 1640 (Gibco, USA) medium supplemented with 10% heat-inactivated fetal calf serum (FCS, HyClone, USA), 100 U/mL penicillin and 100 μg/mL streptomycin (Shanghai, China) in a humidified environment of 95% atmospheric air and 5% CO_2_. To determine the effect of cytokines on cell proliferation and IgA1 production, cells were cultured with IL-17. For dose-dependent experiments, the cells were treated for 48 h with 5–320 ng/mL IL-17. For time-dependent tests, cells were treated with 160 ng/mL IL-17 for 24–72 h (by previous work). Cell proliferation was analyzed with the Cell Counting Kit-8 (CCK-8, China) assay.

To explore the mechanism of aberrant IgA1 glycosylation, cells were cultured in 96-well culture plates at a seeding density of 4 × 10^4^/mL. Then, the cells were treated with 12.5 μg/mL *Escherichia coli* lipopolysaccharide (LPS; Sigma-Aldrich, St. Louis, MO), 160 ng/mL recombinant human IL-17 (R&D Systems, USA), or 1.0 nmol/mL 5-azacytidine (5-AZA; Sigma-Aldrich, St. Louis, MO) + 160 ng/mL IL-17 at a density of 2.65 × 10^5^/mL per well in 6-well culture plates for 48 h. Then, C1GALT1 and Cosmc expression were measured with real time RT-PCR and western blotting. To analyze IgA glycosylation, supernatants were collected. In previous studies, we and others have already demonstrated that LPS suppresses the expression of C1GALT1 and Cosmc [10,11], and 5-AZA is an inhibitor of DNA methylation. We have also reported that de-methylation upregulated Cosmc expression significantly [12]. Therefore, in this study, LPS was used as a positive control, and 5-AZA was used as a negative control.

### 2.2. CCK-8 assay

DAKIKI cells were cultured in 96-well plates at a density of 1 × 10^4^ cells per well for 12 h. Afterwards, cells were starved in serum-free medium for 24 h and then incubated with or without different concentrations of IL-17 for 48 h. Cell proliferation was assessed using the CCK-8 assay (Dojindo, Kumamoto, Japan) according to the manufacturer’s instructions. Briefly, after treatment, 10 μL CCK-8 reagent was added to each well and incubated at 37 °C for 2 h. The optical density was read at a wavelength of 450 nm with a microplate reader (Synergy™ H1, BioTek).

### 2.3. Determination of IgA1 protein levels in supernatants

IgA1 levels in the supernatant of culture wells were measured in duplicate using enzyme-linked immunosorbent assay (ELISA). Briefly and as described previously, 96-well plates were coated with goat anti-human IgA antibody (Southern Biotechnology Associates, Birmingham, AL) overnight at 4 °C [9]. After the plates were blocked, samples were added in duplicate. The plates were incubated overnight at 4 °C and then incubated with horseradish peroxidase-conjugated goat anti-human IgA antibody (Southern Biotechnology Associates, Birmingham, AL) for 2 h at 37 °C. The color was developed with tetramethyl benzidine dilution (TMB) and detected using a Bio-Rad 550 at 450 nm (Synergy™ H1, BioTek, USA).

### 2.4. Enzyme-linked lectin binding assays

IgA1 glycosylation in the supernatant of each culture well was measured using a helix aspersa lectin (HAA) binding assay as previously reported [13]. Briefly, 6-well plates were coated with goat anti-human IgA antibody and blocked as described above. Samples were added to the plates in duplicate and incubated overnight at 4 °C. The IgA1 collected was subsequently desialylated by treatment with neuraminidase from *Vibrio cholerae* (Roche, Penzberg, Germany) for 3 h at 37 °C. Then, the plates were incubated with biotinylated HAA lectin for 3 h at 37 °C, and lectin binding was detected with avidin-horseradish peroxidase conjugate (ExtrAvidin; Sigma-Aldrich, St. Louis, MO). The color was developed and measured as above (Synergy™ H1, BioTek).

### 2.5. RNA extraction and real-time PCR

Total RNA was extracted from cells using Trizol reagent (Invitrogen, Carlsbad, CA). cDNA was synthesized from total RNA using the RevertAid First Strand cDNA Synthesis Kit (Thermo Scientific Fermentas, Vilnius, Lithuania). The resulting cDNA (1 μg) was amplified in real time, with a 20 μL reaction mixture containing SYBR Green PCR Master Mix (Applied Biosystems, Foster City, CA), appropriate primer pairs and water in a Bio-Rad/MJ Chromo4 real-time PCR analyzer (Bio-Rad, Hercules, CA). The samples were incubated at 95 °C for 3 min, followed by 40 cycles with 30 s denaturation at 94 °C, 30 s annealing at 60 °C and 30 s extension at 72 °C. The expression level of target genes was normalized to the reference gene β-actin, which was measured in the same cDNA sample. Sequences of primers used in this study are as follows: C1GALT1: forward, 5′-AAGGTTGACACCCAGCCTAA-3′, reverse, 5′-CTTTGA-CGTGTTTGGCCTTT-3′; Cosmc: forward, 5′-GCTCCTTTTTGAAGGGTGTG-3′, reverse, 5′-TACTGCAGCCCAAAGACTCA-3′; β-actin: forward, 5′-TCACCCA-CACTGTGCCCATCTACGA-3′, reverse, 5′-CAGCGGAACCGCTCATTGCCAAT-GG-3′. All of the reactions were performed in triplicate, and the results were analyzed using the 2^−ΔΔCT^ method.

### 2.6. Protein extraction and Western blot

Western blotting analysis was performed as described previously [14]. The primary antibodies used were anti-C1GALT1 (1:200; Santa Cruz Biotechnology, Santa Cruz, CA), anti-Cosmc (1:200; Santa Cruz Biotechnology, Santa Cruz, CA) and anti-β-actin (1:200; Santa Cruz Biotechnology, Santa Cruz, CA). The relative C1GALT1 and Cosmc protein levels were normalized to β-actin, and the ratio was compared to that of the control group.

### 2.7. Statistical analysis

All of the experiments were performed at least three times and tested for normality. All of the data were presented as the mean ± standard deviation (SD). Statistical differences between groups were evaluated using an independent Student’s *t*-test on SPSS software (version 16.0; SPSS, Chicago, IL). A *p* values of <.05 was considered significant.

## 3. Results

### 3.1. Proliferation of DAKIKI cells incubated with IL-17

**Figure 1.**
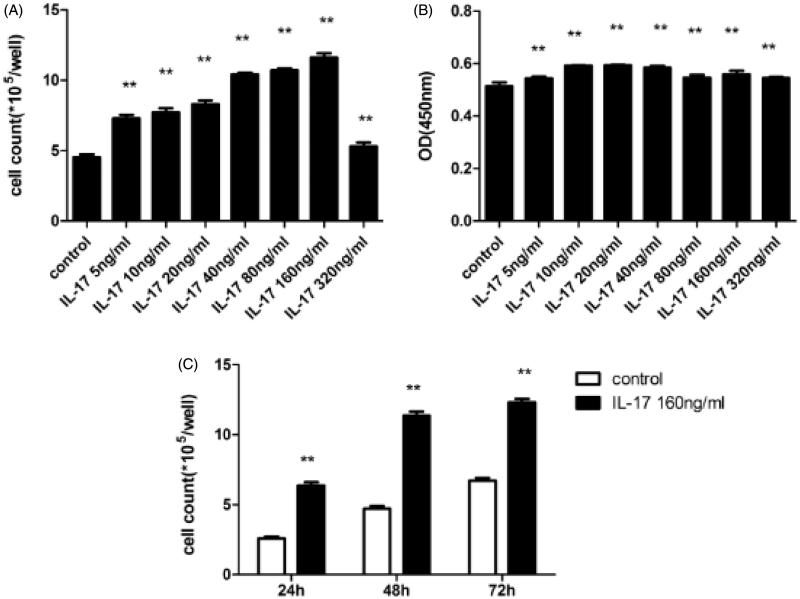
Effect of IL-17 on cell proliferation in DAKIKI cells. (A) In dose-dependent experiments, IL-17 at 160 ng/mL stimulated DAKIKI cell proliferation as measured by manual cell counting. (C) IL-17 (160 ng/mL) time-dependently stimulated DAKIKI cell proliferation as examined by cell counting. (B) In the CCK-8 assay, IL-17 at a concentration of 5-320 ng/mL dose-dependently stimulated DAKIKI proliferation. However, 320 ng/mL IL-17 inhibited proliferation in DAKIKI cells. *p < .05, compared to control; **p < .01, compared to control.

To determine the effects of IL-17 on cell proliferation in DAKIKI cells, dose-dependent and time-dependent experiments were performed. In dose-dependent experiments, 160 ng/mL IL-17 stimulated cell proliferation, whereas 320 ng/mL IL-17 inhibited cell proliferation as assessed by manual cell counting (Figure 1(A)) and the CCK-8 assay (Figure 1(B)). Moreover, at a concentration of 5–160 ng/mL, IL-17 stimulated cell proliferation in a dose-dependent manner when assessed by cell counting manually (Figure 1(A)). Accordingly, 160 ng/mL was chosen as the optimal IL-17 concentration for further studies. In time-dependent experiments, 160 ng/mL IL-17 stimulated cell proliferation in a time-dependent manner as evaluated by manual cell counting (Figure 1(C)).

### 3.2. Production of IgA1 in DAKIKI cells incubated with IL-17

**Figure 2.**
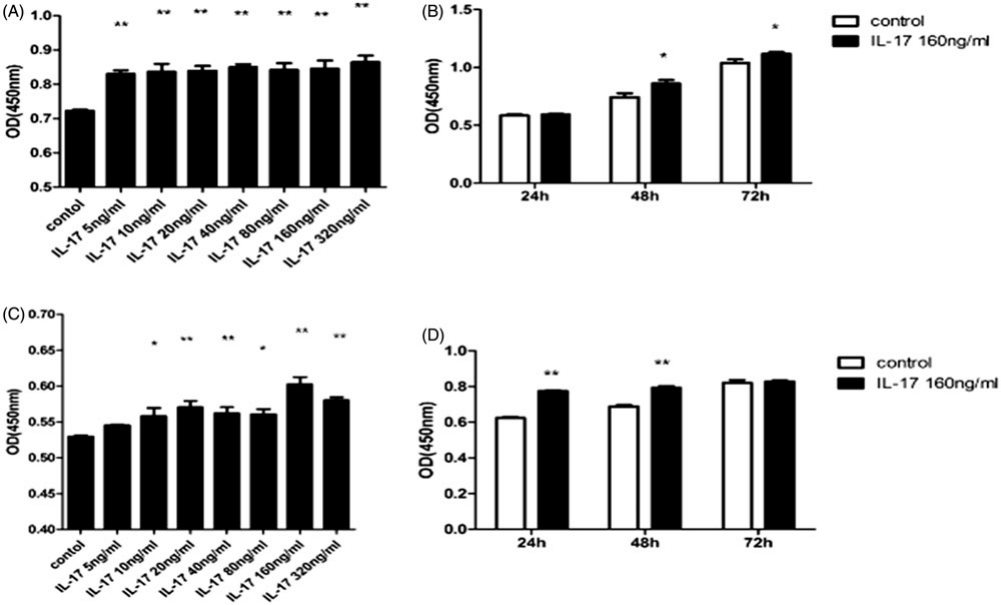
Effect of IL-17 on IgA1 production and underglycosylation in DAKIKI cells. (A) IL-17 at a concentration of 5∼320 ng/mL dose-dependently stimulated IgA1 production in DAKIKI cells. (B) IL-17 at a concentration range of 5∼320 ng/mL dose-dependently stimulated IgA1 underglycosylation in DAKIKI cells. IL-17 (320 ng/mL) had a similar effect, although the IgA1 underglycosylation was lower than that stimulated with 160 ng/mL IL-17. (C) IL-17 (160 ng/mL) time-dependently stimulated IgA1 production in DAKIKI cells. (D) IL-17 (160 ng/mL) time-dependently stimulated IgA1 underglycosylation in DAKIKI cells. *p < .05, compared with the control; **p < .01, compared with the control.

**Figure 3.**
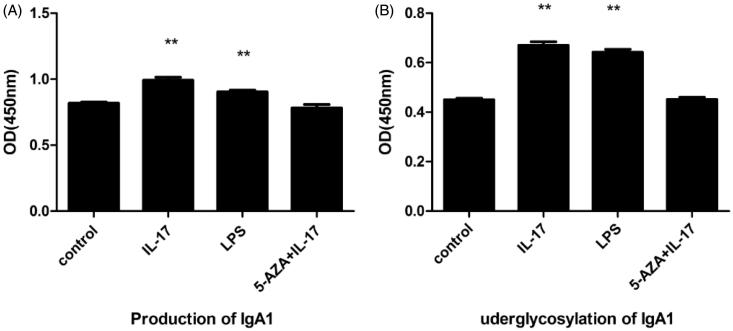
IL-17 and LPS increased the production and underglycosylation of IgA1 in DAKIKI cells. Cells were stimulated with 160 ng/mL of IL-17, while stimulated with 12.5 μg/mL of LPS served as a positive control and 1.0 μmol/L of 5-AZA +160 ng/mL of IL-17 or control as a negative control. (A) IL-17 significantly increased the production of IgA1, compared with negative control, and the effect was stronger than that of LPS. (B) IL-17 obviously promoted the underglycosylation of IgA1, compared with negative control, although the effect was stronger than that of LPS. *p < .05, compared with the control; **p < .01, compared with the control.

In determining the effects of IL-17 on IgA1 production in DAKIKI cells, we found that between 5 and 320 ng/mL IL-17 stimulated IgA1 production. With increasing concentrations of IL-17, IgA1 production increased accordingly (Figure 2(A)). IL-17 at 320 ng/mL had the strongest effect on IgA1 production. Furthermore, 160 ng/mL IL-17 stimulated IgA1 production in a time-dependently between 24 and 72 h (Figure 2(C)). It was observed that IgA1 production was significantly increased with IL-17 treatment compared with the negative control (5-AZA + IL-17), and the effect was stronger than that of LPS (Figure 3(A)).

### 3.3. Underglycosylation of IgA1 in DAKIKI cells incubated with IL-17

When the effect of IL-17 on IgA1 underglycosylation in DAKIKI cells was further examined, we found that IL-17 stimulated the IgA1 underglycosylation at a concentration range of 5–320 ng/mL. Although it inhibited cell proliferation, IL-17 at 160 ng/mL had a much stronger effect on IgA1 underglycosylation than IL-17 at 320 ng/mL (Figure 2(B)). In time-dependent experiments, IL-17 at 160 ng/mL stimulated IgA1 underglycosylation between 24 and 72 h (Figure 2(D)). It was observed that IgA1 underglycosylation was significantly increased with IL-17 treatment compared with the negative control (5-AZA + IL-17), and the effect was stronger than that of LPS (Figure 3(B)).

### 3.4. Effect of IL-17 on C1GALT1 and Cosmc protein and mRNA levels in DAKIKI cells

**Figure 4.**
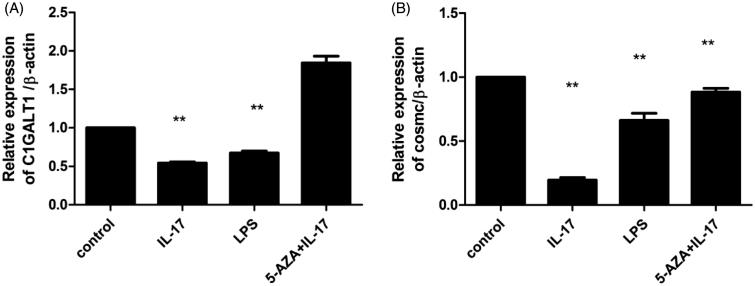
IL-17 decreased the mRNA expression of C1GALT1 and Cosmc in DAKIKI cells. Cells were stimulated with 160 ng/mL of IL-17, while stimulated with 12.5 μg/mL of LPS served as a positive control and 1.0 μmol/L of 5-AZA +160 ng/mL of IL-17 or control as a negative control. (A) IL-17 can decreased the mRNA expression of C1GALT1, compared with negative control, although the effect was stronger than that of LPS. (B) IL-17 significantly decreased the mRNA expression of Cosmc, compared with negative control, although the effect was stronger than that of LPS. *p < .05, compared with the control; **p < .01, compared with the control.

**Figure 5.**
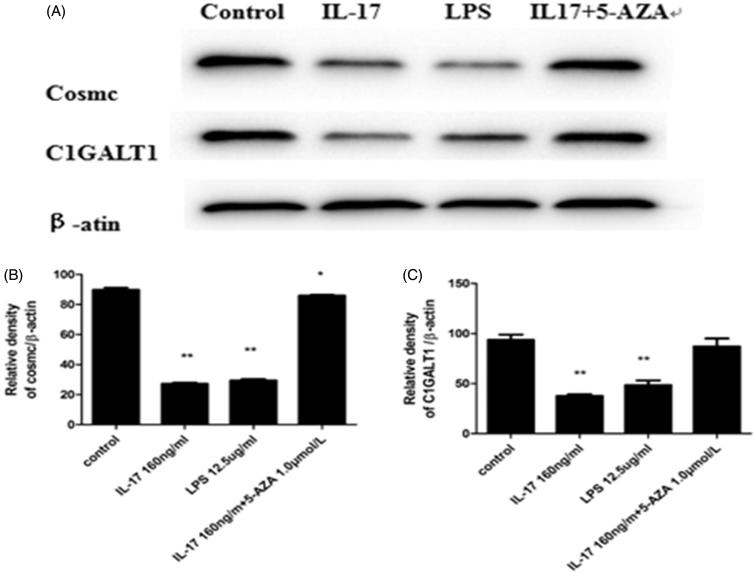
IL-17 decreased C1GALT1 and Cosmc protein expression in DAKIKI cells. Cells were stimulated with 160 ng/mL IL-17. Stimulation with 12.5 (g/mL LPS was the positive control, and 1.0 μmol/L 5-AZA+160 ng/mL IL-17 or medium was the negative control. (A, B) IL-17 decreased Cosmc protein expression compared with the negative control, although the effect was stronger than that of LPS. (A, C) IL-17 significantly decreased C1GALT1 protein expression compared with the negative control, although the effect was stronger than that of LPS. *p < .05 compared with control; **p < .01 compared with control.

To examine the mRNA expression levels of C1GALT1 and its chaperone Cosmc in DAKIKI cells treated with IL-17, real-time RT-PCR analysis was performed. C1GALT1 mRNA levels were significantly decreased following treatment with 160 ng/mL IL-17 compared to the medium control and the 5-AZA + IL-17 control. However, the effect was less intense than the positive control of 12.5 μg/mL LPS (Figure 4(A)). The levels of Cosmc mRNA exhibited a similar trend as that observed for C1GALT1 (Figure 4(B)). To assess the C1GALT1 and its chaperone Cosmc protein levels in DAKIKI cells treated with IL-17, western blotting analysis was used. Compared with the medium control and the 5-AZA control, C1GALT1 protein expression was significantly decreased with 160 ng/mL IL-17; however, in the 12.5 μg/mL LPS positive control, C1GALT1 expression increased more significantly (Figure 5(A–C)). Cosmc protein expression exhibited a similar trend as C1GALT1 (Figure 5(A–C)).

## 4. Discussion

Lymphocyte functions, including cytokine profiles, have been investigated extensively in IgAN patients, and several abnormalities have been identified [15]. In recent studies [16,17], polymeric IgA molecules generated by the activity of polyclonal B lymphocytes cells have been observed in IgA nephropathy patients. B lymphocytes cells are secretory IgA cells regulated by T lymphocytes cells. When the immune regulation function of T lymphocytes cells becomes compromised, B lymphocytes cells might produce excessive amounts of IgA. It is widely accepted that Th17 lymphocytes play a crucial role in the pathogenesis of autoimmune diseases by promoting chronic inflammatory responses. The function of Th17 cells is mainly achieved through IL-17 function. IL-17 release at inflammation sites such as the kidneys and the skin can amplify the immune response by increasing the influx of effector cells. Furthermore, IL-17 can increase B-cell activation and antibody production, contributing to overactivation of the B-cell compartment. IL-17 is considered a multifunctional cytokine that has been reported to be involved in inflammation and autoimmune diseases such as primary nephritic syndrome [18], systemic lupus erythematosus [19] and rheumatoid arthritis [20]. Therefore, whether IL-17 participates in the immune pathogenesis of IgAN has not been elucidated. In this study, we found that IL-17 stimulated cell proliferation in the B-cell line DAKIKI. And the highest dose of IL-17(320 ng/mL) have an inhibitory effect on cell proliferation, the reason perhaps is because the highest dose of IL-17 is poisonous to the cells. We further demonstrated that IL-17 promoted IgA1 production in the IgA1-producing cell line DAKIKI, which at least in part might be caused by the increase in cell number from increased cell proliferation.

Aberrant IgA1 glycosylation plays a vital role in the pathogenesis of IgA nephropathy. Abnormal expression or activity of C1GALT1 and Cosmc might result in aberrant protein glycosylation. Hashimoto et al. have demonstrated that aberrantly glycosylated IgA might affect the formation of macromolecular IgA, including IgA-IgG immune complexes and subsequent complement activation, leading to full progression of IgAN [21]. In peripheral blood mononuclear cells (PBMC), tonsil tissue and tonsillar B-cells from IgAN patients, C1GALT1 and Cosmc gene and protein expression are significantly downregulated [13,22]. Moreover, C1GALT1 expression in tonsillar B-cells from IgAN patients is notably correlated with clinical characteristics such as estimated glomerular flow rate (GFR), proteinuria and histologic injury score [22]. Cosmc mRNA expression and IgA1 O-glycosylation levels in IgAN patients were significantly lower than they were in normal controls. Treatment with LPS might inhibit Cosmc expression and increase the IgA1 secretion in the peripheral lymphocytes of IgAN patients, which would result in a significant increase in levels of aberrantly glycosylated IgA1 [23]. However, 5-AZA is an inhibitor of DNA methylation. We found that it might enhance the methylation modifications that LPS inhibits, such as Cosmc expression. Moreover, de-methylation could effectively reverse the repressed Cosmc mRNA expression [12]. In this study, we showed that IL-17 decreased C1GALT1 and Cosmc expression in the B-cell line DAKIKI; these genes might contribute to IgA1 underglycosylation. Therefore, the mechanism of aberrant IgA1 glycosylation induced by IL-17 might be caused by abnormal epigenetics regulation. Otherwise, in recent years research [24,25] also found that the concentration of serum sST2 was positively correlated with the levels of IL-10 in IgAN patients and increased sST2 and IL-10 may be involved in the pathogenic process of IgAN and IL-17, IL-10, IL-6 and other cytokines seem to have a central role in inflammation and progression of kidney injury, also involved in the pathogenesis on IgAN. It need more further studies to research the relationship between cytokines in progression of IgAN.

**Figure 6.**
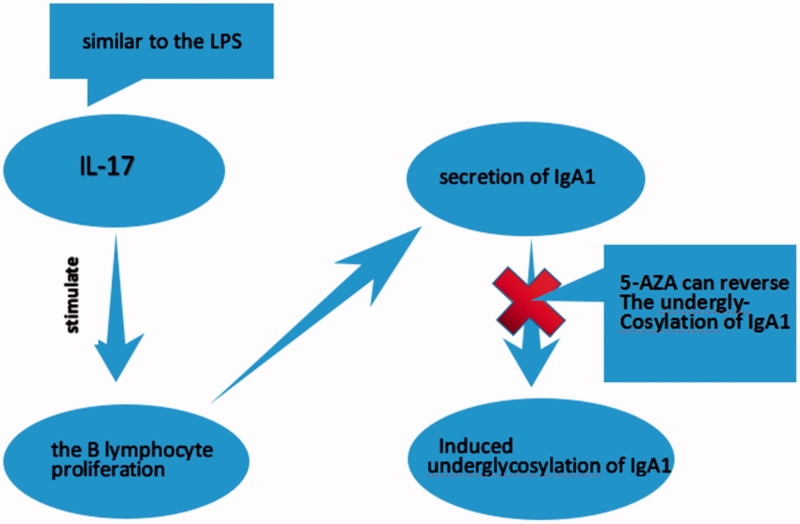
Our findings about the effects of IL-17 *in vitro* studies.

In summary, we demonstrated that IL-17 promoted B-cell proliferation and IgA1 production in DAKIKI cells. IL-17 induced IgA1 underglycosylation in a dose-dependent and time-dependent manner. The molecular mechanism causing IgA1 underglycosylation by IL-17 might be similar to LPS. Both treatments lead to decreased expression of C1GALT1 and Cosmc. Furthermore, 5-AZA can reverse IgA1 underglycosylation, which suggests that abnormal methylation might be involved in regulating genes related to glycosylation. These results suggested that IL-17 might play an important role in IgAN pathogenesis, and modulation of immune functions by IL-17 might be a target to prevent IgAN recurrence in the future (Figure 6).
